# Frequency and Causes of False-Positive Elevated Plasma Concentrations of Fasting Gut Hormones in a Specialist Neuroendocrine Tumor Center

**DOI:** 10.3389/fendo.2020.606264

**Published:** 2020-12-16

**Authors:** Olivia L. Butler, Monica M. Mekhael, Arslan Ahmed, Daniel J. Cuthbertson, D. Mark Pritchard

**Affiliations:** ^1^ School of Medicine, University of Liverpool, Liverpool, United Kingdom; ^2^ Insitute of Lifecourse and Medical Sciences, University of Liverpool, Liverpool, United Kingdom; ^3^ ENETS Centre of Excellence, Liverpool University Hospitals NHS Foundation Trust, Liverpool, United Kingdom; ^4^ Institute of Systems, Molecular and Integrative Biology, University of Liverpool, Liverpool, United Kingdom

**Keywords:** gut hormones, somatostatin, glucagon, neuroendocrine neoplasia, false positive

## Abstract

**Introduction:**

In the UK, the fasting plasma concentrations of a panel of gut hormones (comprising vasoactive intestinal peptide (VIP), gastrin, pancreatic polypeptide (PP), glucagon, somatostatin and chromogranin A) are measured to evaluate patients who have or who (due to unexplained and compatible symptoms) are suspected of having neuroendocrine tumors (NETs). False positive elevated hormone concentrations are sometimes found.

**Objective:**

To evaluate the frequency and implications of false positive fasting gut hormone results.

**Methods:**

Retrospective audit of fasting gut hormone profile results at a large UK university teaching hospital over 12 months.

**Results:**

Fasting gut hormone concentrations were measured in 231 patients during 2017. No NETs were found in the 88 patients who had this test performed only to investigate symptoms. 31 false positive gastrin, 8 false positive chromogranin A, two false positive glucagon, three false positive somatostatin, one false positive PP, and one false positive VIP results were found. We extended the audit for glucagon and somatostatin for an additional two years and found seven probable false-positive raised glucagon concentrations and four probable false-positive elevated plasma somatostatin concentrations in total.

**Conclusions:**

False-positive elevations of plasma gastrin and chromogranin A were common and causes such as proton pump inhibitor use or inadequate fasting accounted for most cases. Elevated plasma concentrations of the other gut hormones were also detected in patients who had no other evidence of NET. Other diagnoses (e.g. cirrhosis and medullary thyroid carcinoma for hypersomatostatinemia and type 2 diabetes mellitus, pancreatitis, liver or renal impairment for hyperglucagonemia) may cause these false positive results.

## Introduction

Neuroendocrine tumors (NETs) comprise a diverse set of neoplasms that can arise at various anatomical sites (1). NETs can present with diverse symptoms and many cases are also detected incidentally during investigations for unrelated medical conditions (2). Although many NETs are non-functional (i.e. they do not secrete hormones), a significant minority secrete hormones and peptides resulting in classical functional syndromes of hormone excess. With the exception of carcinoid syndrome, which is most frequently associated with liver metastases from a primary small bowel NET, most other functional NETs arise in the foregut, especially the pancreas. Such tumors can secrete a range of hormones (usually in isolation) such as insulin, gastrin, somatostatin, glucagon or vasoactive intestinal peptide (VIP) and excess secretion of these hormones may result in classical syndromes (3). For example, gastrinomas result in overproduction of gastric acid and Zollinger-Ellison syndrome, somatostatinomas are classically associated with the triad of type 2 diabetes mellitus, cholelithiasis and diarrhea while glucagonomas can cause the necrolytic migratory erythema skin rash, type 2 diabetes mellitus and weight loss (3, 4). Most pancreatic NETs develop sporadically, but a minority are associated with genetically inherited syndromes such as multiple endocrine neoplasia type 1 (MEN1), von Hippel Lindau syndrome or neurofibromatosis type 1 (3).

Patients who are suspected or confirmed as having a NET usually undergo comprehensive biochemical and radiological investigations to fully characterize the primary site and extent of metastatic spread of their tumors (5, 6). The results of these investigations influence the choice of treatment and also provide prognostic information. One component of the diagnostic evaluation of a patient with a possible NET is to perform a biochemical assessment of its functional status (3). Most patients also usually undergo cross-sectional and functional imaging investigations (e.g. CT/MR scan, Octreoscan or ^68^Ga DOTA-peptide PET/CT) and histological confirmation of the diagnosis and tumor grading. The North American and European neuroendocrine tumor societies (NANETS and ENETS) have published guidance on the appropriate use of biochemical tests in various scenarios, particularly the assessment of potential functional pancreatic NETs. In many countries therefore, clinicians request analysis of the concentrations of individual gut hormones according to their clinical suspicion. However, the situation in the UK is somewhat different, in that the vast majority of hospitals send their samples to be analyzed at a central supraregional accredited reference laboratory in London. This laboratory performs a ‘fasting gut hormone profile’, which measures the plasma concentration of the general NET biomarker, chromogranin A together with the plasma concentrations of a number of enteroendocrine hormones namely gastrin, somatostatin, glucagon, pancreatic polypeptide (PP) and vasoactive intestinal peptide (VIP). Thus, even if the clinician is only interested in measuring the concentration of an individual hormone (e.g. gastrin in a patient with suspected Zollinger-Ellison syndrome), the results of the whole gut hormone profile are provided in the majority of cases. The rationale may be that in certain conditions such as MEN1, the concentrations of more than one gut hormone may be simultaneously elevated. Other biochemical investigations such as measurement of the plasma or 24 h urinary concentration of 5-HIAA may also be undertaken, but these tests are distinct from the ‘fasting gut hormone profile’ and are usually performed at each base hospital rather than at the central facility.

In addition to its use in the assessment and monitoring of patients who have confirmed NETs, the fasting gut hormone profile is also used indiscriminately by some clinicians to investigate whether unexplained symptoms such as persistent diarrhea, facial flushing or ‘dizzy spells’ are caused by functional NET syndromes. This is despite symptoms such as flushing having a myriad of different NET and non-NET related causes (7). The positive predictive value of the fasting gut hormone test in this setting is consequentially very low. Therefore, it is not uncommon for patients to be found to have elevated concentrations of one or more gut hormones and to undergo additional investigations to evaluate whether they have a NET. Such investigations can be potentially invasive, expensive and psychologically distressing and their results are frequently unremarkable (i.e. the elevated gut hormone concentration is eventually classified as a false-positive result).

We have recently observed an increasing volume of referrals to the NET multidisciplinary team at Liverpool ENETS Centre of Excellence to investigate patients who had been found to have abnormal fasting gut hormone profiles, especially when these tests had been ordered by non-NET specialist clinicians to investigate a variety of non-specific symptoms. We therefore set out to determine the frequency of such false positive-fasting gut hormone test results (i.e. in those patients who were subsequently found to have no evidence of a NET) at our specialist NET center, the extent to which these biochemical results led to additional investigations and to elucidate whether there were any potential explanations for the abnormal test results.

## Methods

We conducted a retrospective audit using electronic patient records at the Royal Liverpool University Hospital, Liverpool, UK. This is a large tertiary adult general hospital and also manages patients within Liverpool’s European Neuroendocrine Tumour Society (ENETS) Centre of Excellence. Every patient who had a fasting gut hormone test performed between 1st January 2017 and 31st December 2017 was identified from an electronic database. Information was collected regarding patient demographics, clinical indication for the test and diagnoses from histology, radiology and biochemistry reports and electronic patient records. The project did not require ethical approval, but was registered and approved by the hospital’s audit department.

We subsequently extended the audit to include the 3 year period between August 2016 and August 2019 using the same methodology specifically to evaluate all patients who had elevated concentrations of glucagon or somatostatin. We explored alternative potential causes for the elevated concentrations of these hormones in those patients who did not have a confirmed NET.

Fasting plasma gut hormone assays were analyzed by radioimmunoassay at the Department of Molecular Endocrinology, Hammersmith Hospital and at Charing Cross Hospital, London. These radioimmunoassays were developed and validated in the academic laboratory of Prof S.R. Bloom over several decades and are regarded by many investigators as the gold standard assays for these hormones. The methods employed are described in (8, 9). Details of the individual radioimmunoassays used and their performances can be found in the following publications: Gastrin: primary antibody Gas 8 1:10 (10); Glucagon: primary antibody RCS5 1:50 (11); Pancreatic polypeptide: primary antibody HPP/CCK5 1:50 (12, 13); VIP: primary antibody V9 1:100 (14, 15); Somatostatin: primary antibody K2 1:10 (16, 17); Chromogranin A: SAS radioimmunoassay (18, 19). The assays are performed in a fully accredited national UK National Health Service reference laboratory which is subject to stringent regular audit and quality control measures. Results of gut hormones were deemed to be abnormal if they met any of the following criteria: gastrin >40pmol/L, glucagon >50 pmol/L, pancreatic polypeptide (PP) >300 pmol/L, VIP >30 pmol/L, somatostatin >150 pmol/L, or Chromogranin A >60 pmol/L.

## Results

### Cohort Characteristics

Fasting gut hormone profiles were measured in 231 patients [135 female (58%)], median age 63 years (range 18–92 years) during 2017. Four patients had repeated tests within the study timeframe, therefore 235 test results were evaluated. Tests were requested by a variety of clinicians, the most frequent requesting specialties being gastroenterology (48%), general surgery (24%) and endocrinology (18%).

Tests were performed with varying levels of pre-investigation probability of having a NET: 61 (26%) patients had a previously diagnosed NET and the test was performed for surveillance, seven (3%) patients had MEN1 and underwent fasting gut hormone testing for surveillance of this condition and 75 (32%) patients were tested because of a strong radiological suspicion of a NET (e.g. the presence of a hypervascular pancreatic tumor or hypervascular liver metastases compatible with NET with no primary tumor site identified) or histopathological detection of a NET on a biopsy or surgical resection specimen. Of these 143 patients, 136 were eventually confirmed to have a NET and a NET was excluded in the other seven patients (most of whom were being investigated because of a radiological abnormality). The majority of these NETs (71%) arose in the foregut, as expected by the hormones that are tested in the “fasting gut hormone profile” (50% pancreatic, 13% gastric, and 8% duodenal). In a few patients, the test was performed due to an unknown primary site and the commonest other primary site was small bowel (21%). The remaining 88 (39%) patients underwent fasting gut hormone testing on account of unexplained symptoms for which the referring clinician considered NET to be within the differential diagnosis; these patients had a wide range of symptoms with diarrhea (33%) and flushing (14%) being the most common. The vast majority of these tests (88%) were requested by clinicians who were not core members of the hospital’s NET multidisciplinary team. None of these 88 patients was subsequently confirmed to have a NET, although the extent of subsequent investigations was highly variable.

### Fasting Gut Hormone Results

Within the whole cohort of 231 patients, elevated gastrin concentrations were detected in 75 patients, chromogranin A in 35, glucagon in 13, somatostatin in five, PP in three, and VIP in two patients. Some patients had multiple elevated plasma hormone concentrations, the most frequent combination being raised gastrin and chromogranin A (n=7).

We next assessed what proportion of those patients who had elevated concentrations of each hormone eventually had a confirmed NET diagnosis and found that 31/75 patients with elevated gastrin, 8/35 elevated chromogranin A, 2/13 elevated glucagon, 3/5 elevated somatostatin, 1/3 elevated PP, and 1/2 elevated VIP concentrations had no other biochemical or radiological evidence to confirm the presence of a NET (**Table 1**). The anatomical sites of the primary NETs that were associated with true positive elevated concentrations of each hormone are also shown in **Table 1**.

**Table 1:** Association of elevated fasting gut hormone parameters with the presence of neuroendocrine tumor (NET).

	Total number of elevated tests	Number of true positive tests in patients who had a confirmed NET	Primary sites of NET in patients who had true positive elevated concentrations of each hormone	Number of false-positive tests (number with concentrations > 2× upper limit of normal)
Gastrin	75	44	18 pancreas 14 gastric 6 duodenal 4 small bowel 1 lung 1 unknown primary	31 (16)
Glucagon	13	11	10 pancreas 1 small bowel	2 (1)
Somatostatin	5	2	2 pancreas	3 (2)
Pancreatic polypeptide (PP)	3	1	1 small bowel	2 (1 non-fasting and spurious) (2)
Vasoactive intestinal peptide (VIP)	2	1	1 pancreas	1 (non-fasting and spurious) (1)
Chromogranin A	35	27	13 small bowel 8 pancreas 2 gastric 1 duodenal 1 lung 2 unknown primary	8 (2)

### Degree of Hormone Elevations in True and False Positive Cases

We next investigated whether the extent to which positive gut hormone results were elevated correlated with whether they represented true positive or false positive findings. When we analyzed all 35 patients who had elevated chromogranin A concentrations, we found significantly higher median concentrations in the 27 patients who had a NET (213pM) (i.e. true positive cases) compared to the eight patients who had no evidence of NET (77pM) (i.e. false positive cases) (p<0.01, Mann Whitney U test). Only two patients who had no evidence of a NET had chromogranin A levels that were elevated more than two times the upper limit of normal. However, we did not find any such correlations for the other gut hormones, although in many cases the numbers of patients in each group were too small to permit meaningful statistical analysis. For gastrin, glucagon, somatostatin, PP and VIP, the median concentrations of the elevated hormones were not significantly different between those patients who had true positive and false positive elevations of these tests. In this cohort therefore, the degree to which the concentrations of gut hormones other than chromogranin A were elevated did not provide a reliable method to distinguish whether it was likely to represent a true positive or false positive result.

### Extension of the Audit for an Additional Two Years Found Additional Cases of False Positive Hyperglucagonemia and Hypersomatostatinemia

In many patients, the causes of the false positive elevations of gut hormones were readily apparent. For example, false positive elevated concentrations of gastrin and chromogranin A frequently resulted from the use of acid suppressing medications and we found that tests being performed under non-fasting conditions accounted for most of the small number of false positive elevated concentrations of both PP and VIP. We are unfortunately not able to attribute a final cause for the hypergastrinemia that was found in 31 patients who had no evidence of NET, as many patients did not have OGD to assess for the presence of atrophic gastritis and/or *H. pylori* infection. However, 61% of these patients were taking a proton pump inhibitor, a well-documented cause of hypergastrinemia, and that is likely to provide the underlying explanation in most of these cases.

However, our findings of false positive elevations of glucagon and somatostatin were more surprising and the causes of such abnormalities are generally less well appreciated. We therefore extended our audit to include an additional two years to see whether we could detect any additional patients at our hospital who had evidence of false positive elevations of glucagon or somatostatin.

The audit was therefore extended to cover the three year period between August 2016 and August 2019. 641 fasting gut hormone tests were performed during this time (including some patients who had multiple tests). We found a total of 38 patients who had elevated plasma concentrations of glucagon, seven of whom had no confirmed evidence of NET and nine patients who had elevated plasma concentrations of somatostatin, four of whom had no confirmed evidence of NET (**Tables 2** and **3**). Most of the patients who were found to have elevated plasma glucagon concentrations, but who were not already known to have a NET, had only marginally elevated levels (only one had a concentration that was more than two times the upper limit of normal) (**Table 2**). Many of these patients were therefore not referred for comprehensive investigations to exclude glucagonoma. Two of these patients also had elevated chromogranin A results, but these were probably caused by co-existing end stage renal failure. We unfortunately cannot therefore definitively exclude the presence of small pancreatic glucagonomas in all these cases, but that diagnosis is considered to be unlikely in most cases. Most of the patients who had elevated plasma somatostatin concentrations in the absence of a confirmed NET did undergo reasonably extensive additional investigations and these tests ruled out a diagnosis of pancreatic NET with a high degree of probability in all cases.

**Table 2:** Clinical details of patients with raised plasma glucagon concentrations.

Patient number	Gender	Age (years)	Plasma glucagon (pM)(N<50pM)	Indication for test	Comorbidities	Additional tests which showed no evidence of NET
1	Male	73	56	Diarrhea	Refractory coeliac disease, diffuse large B cell lymphoma	CT, MR
2	Male	65	68	Diarrhea	*C. difficile* colitis End stage renal failure	CT (CgA elevated but attributed to CKD)
3	Female	39	70	Diarrhea	Type 2 diabetes, obesity, Nonalcoholic fatty liver disease	CgA, US pancreas
4	Male	59	77,74	Diarrhea	Type 2 diabetes, end stage renal failure, *C. difficile* colitis	No imaging, CgA elevated but attributed to CKD
5	Female	63	53	Sweats	High output ileostomy following panproctocolectomy	CT, CgA
6	Female	66	379, 241, 456	Diarrhea	Ulcerative colitis, type 2 diabetes, chronic pancreatitis	CT, MR, CgA, EUS, octreoscan
7	Female	69	62	Flushing	Small bowel intussusception	CgA, repeat glucagon 8pM, CT, capsule enteroscopy

CT, computerised tomography scan; MR, magnetic resonance scan; US, ultrasound scan; EUS, endoscopic ultrasound scan; CgA, chromogranin A; CKD, chronic kidney disease.

**Table 3:** Clinical details of patients with raised plasma somatostatin concentrations.

Patient number	Gender	Age (years)	Plasma somatostatin (pM) (N<150pM)	Indication for test	Comorbidities	Additional tests which showed no evidence of NET
1	Male	43	156	Flushing, family history of NET	Alcoholic liver disease	CT, CgA, urinary 5-HIAA, colonoscopy, OGD
2	Female	73	197 and 473	Non-healing peptic ulcer	Liver transplant	CgA, CT, OGD
3	Female	65	348 and 407	Diarrhea	Bile acid diarrhea, breast cancer	CgA, CT, ^68^Ga-DOTANOC PET/CT, OGD, colonoscopy
4	Female	48	175	Vomiting	Parenteral nutrition for recurrent vomiting	CgA, CT, OGD

CT, computerized tomography scan; OGD, esophagogastroduodenoscopy; CgA, chromogranin A.

30 of the remaining “true positive” 31 patients who had elevated plasma glucagon concentrations had evidence of pancreatic NETs. Four of these patients also had MEN1. The median concentration was 80pM (range 51-1809pM) with 12 of the 30 patients having concentrations more than two times the upper limit of normal, five of whom had concentrations more than 10 times the upper limit of normal. Only the three patients with glucagon concentrations >1,000 pM showed any of the classical symptoms associated with glucagonoma. It is therefore likely that the majority of the patients who had only marginally elevated glucagon concentrations had non-functional pancreatic NETs that were not secreting sufficient glucagon to result in clinical manifestations. One patient who had raised plasma glucagon levels (71 pM) had a small bowel NET, so this too may have been a false positive result. Nonetheless we have classified all these 31 patients as having true positive elevations of plasma glucagon. The five patients who had true positive elevated plasma somatostatin concentrations all had pancreatic NETs, but none of them had confirmed MEN1. In all five cases, the degree of increase was less than 1.5 times the upper limit of normal (median 193 pM, range 161–221 pM) and none presented with symptoms associated with the classical “somatostatinoma syndrome.” These are therefore more likely to be non-functional pancreatic NETs with minimal elevation of plasma somatostatin concentration and do not appear to have been secreting sufficient somatostatin to cause clinical manifestations.

## Discussion

This study has two main conclusions. Firstly, none of the 88 patients who underwent fasting gut hormone testing to evaluate whether their non-specific symptoms were due to a functional NET syndrome was eventually confirmed to have a NET. This suggests that the use of the fasting gut hormone blood test is probably inappropriate in this clinical scenario. As functional pancreatic NETs such as somatostatinomas, glucagonomas, PPomas and VIPomas are very rare (estimated incidences 1 in 10–40 million individuals per year), such an investigation strategy is also likely to result in some false positive test results. These will mean that the patient undergoes additional investigations, which are expensive, but may also provoke anxiety and significant psychological distress for patients and have cost and resource implications for the NHS.

The second main finding of our study was that 47 of 133 (35%) elevated fasting gut hormone test results at our hospital in 2017 occurred in patients in whom there was no other evidence of a NET. The most frequently elevated hormones (39 of these 47 cases) were gastrin and chromogranin A, but only 18 of the 39 patients who had false positive elevations of either of these hormones showed an increase of more than two times the upper limit of normal. The causes of false positive elevations of gastrin and chromogranin A have previously been well documented. Hypergastrinemia commonly results from hypochlorhydria as a result of gastric pathology (autoimmune or *H. pylori* associated atrophic gastritis), surgery (vagotomy) or medication (proton pump inhibitor or H2 receptor antagonist use), but can also be associated with other conditions including *H. pylori* infection *per se*, renal failure and retained gastric antrum after partial gastrectomy (20). Esophagogastroduodenoscopy (OGD) may be required to evaluate the cause of hypergastrinemia and to assess for the presence of type I gastric neuroendocrine tumors in any patients who have autoimmune atrophic gastritis. Elevated circulating concentrations of chromogranin A can be found in renal and hepatic impairment, chronic atrophic gastritis, proton pump inhibitor use and inflammatory bowel disease (4).

Although rare, we also found potential false positive elevations of some of the other gut hormones within our cohort. False positive elevations of PP and VIP were extremely rare and two of the three identified cases were spurious as a result of the test being performed in the non-fasting state. However, we also detected five patients in whom hyperglucagonemia and hypersomatostatinemia did not appear to be associated with a NET. We therefore extended our audit to search for additional potential cases of false positive hyperglucagonemia and hypersomatostatinemia over a longer three year audit period. In total, we found four cases of probable false positive hypersomatostatinemia (two of whom had somatostatin concentrations more than two times the upper limit of normal on at least one occasion) and seven cases of probable false positive hyperglucagonemia (only one of whom had a glucagon concentration more than two times the upper limit of normal).

Somatostatinomas are classically associated with a clinical triad of diabetes mellitus, cholelithiasis and diarrhea, collectively termed “somatostatinoma syndrome.” However most patients only have partial manifestation of this triad (21). Somatostatinomas are very rare (incidence approximately 1 in 40 million persons per year) and approximately 45% are thought to arise in patients with MEN1 (3). Patients with glucagonoma can present with necrolytic migratory erythema (67%–90%), glucose intolerance (38%–87%) and weight loss (66%–96%) (3). This tumor is also very rare (approximately 1 in 20 million persons per year) and up to 20% cases are thought to be associated with MEN1 (3).

Several studies have suggested that elevated circulating concentrations of somatostatin can be found in patients who have cirrhosis (22–26) or medullary carcinoma of the thyroid (27–29). A single study also described elevated concentrations in patients with hypothyroidism (30). There is also some evidence to suggest that circulating somatostatin concentrations can be increased in diabetes mellitus (31, 32) ulcerative colitis (33) and vascular dementia (34). Studies have also confirmed that elevated somatostatin concentrations are not associated with a range of other conditions including autosomal dominant polycystic kidney disease (35), chronic gastritis (36), biliary stone disease (37), acromegaly (38), obesity (39, 40), chronic pancreatitis (41) and irritable bowel syndrome (42). The published literature has also suggested that circulating somatostatin concentrations tend to increase with age (43, 44), but other physiological variables such as gender and ethnicity do not seem to have a major influence on this parameter (although there appear to have been few studies that have systematically addressed this).

Liver cirrhosis and medullary carcinoma of the thyroid therefore appear to be the two main conditions for which several studies have reported an association with elevated fasting plasma somatostatin concentrations. We note that two of the four patients in whom we identified false positive elevated somatostatin concentrations had current or previous severe chronic liver disease (**Table 2**). Circulating calcitonin or carcinoembryonic antigen (CEA) concentrations were not measured in any of our patients, so we cannot definitively exclude a diagnosis of medullary carcinoma of the thyroid in the other two patients. However, none of the patients have developed other signs of this disease during at least two years follow up.

We found little evidence to suggest that physiological variants such as age, gender and ethnicity have a significant effect upon circulating glucagon concentrations (45). We did however identify documented associations between elevated circulating glucagon concentrations and a range of medical conditions. These include diabetes mellitus (46–48) [including gestational diabetes (49)], acute and chronic pancreatitis (50, 51), chronic kidney disease (including that associated with type 2 diabetes mellitus) (52, 53) and liver cirrhosis (54–56). Small studies have also suggested potential associations with hyperthyroidism (57) and the second trimester of pregnancy (57), although these findings have not yet been independently verified to the best of our knowledge.

There is therefore reasonable quality data from more than one independent study to suggest that diabetes mellitus, pancreatitis, chronic kidney disease and cirrhosis may be potential reasons for elevated circulating glucagon levels in the absence of a glucagonoma. Of the seven patients that we identified who had potential false positive fasting plasma glucagon concentrations, three had diabetes mellitus, two had end stage renal failure and one had chronic pancreatitis (**Table 3**). Plausible explanations for hyperglucagonemia were therefore found in four of these seven patients, and the remaining three patients all had fasting glucagon concentrations (56, 53, 62 pM) that were only minimally above the upper limit of normal for the assay (50pM).

One limitation of our study is that not all patients who had abnormal results were referred to the NET multidisciplinary team for full assessment. Thus, not all patients had comprehensive investigations such as ^68^Ga DOTA-peptide PET/CT scans or endoscopic ultrasound scans to exclude very small pancreatic or duodenal NETs. However only two of the eleven patients that we identified as having probable false positive elevated glucagon or somatostatin concentrations did not have at least a CT scan, and all patients who had hypersomatostatinemia underwent esophagogastroduodenoscopy, so it is unlikely that many occult NETs have been missed in this cohort. A further limitation is that several patients did not undergo repeat testing (particularly after advice about strict adherence to fasting) to exclude spurious results.

In conclusion, this study suggests that the fasting gut hormone test is probably used inappropriately by some clinicians when it is ordered to investigate whether symptomatic patients have rare functional NET syndromes. When this panel of tests is performed in isolation, it also appears to frequently generate results that are potentially unhelpful and difficult to interpret. We therefore recommend that elevations of gut hormone concentrations in patients who are not already known to have a NET should initially be confirmed by repeating the test and making sure that the patient has been adequately fasted before any further investigations are undertaken. Persistently elevated results, especially when these are more than two times the upper limit of normal usually require further investigations (e.g. radiological imaging) to evaluate for the presence of a NET. Clinicians who order the fasting gut hormone test should also be aware that a number of other medical conditions (e.g. liver and renal impairment) or drugs (especially proton pump inhibitors causing hypergastrinemia) can potentially cause elevated concentrations of the various components of the gut hormone profile. The presence of such conditions may influence whether additional potentially expensive or invasive investigations should be considered.

## Data Availability Statement

The raw data supporting the conclusions of this article will be made available by the authors, without undue reservation.

## Ethics Statement

Ethical review and approval was not required for the study on human participants in accordance with the local legislation and institutional requirements. Written informed consent for participation was not required for this study in accordance with the national legislation and the institutional requirements.

## Author Contributions

OB performed data collection and analysis. MM and AA performed systematic literature reviews, and all these three authors as well as DC reviewed the manuscript for important intellectual content. DMP supervised the study, analyzed the data, and wrote the first draft of the manuscript for publication. All authors contributed to the article and approved the submitted version.

## Conflict of Interest

DMP has had consultancies with Ipsen and Advanced Accelerator Applications and has received research funding from Trio Medicines Ltd.

The remaining authors declare that the research was conducted in the absence of any commercial or financial relationships that could be construed as a potential conflict of interest.
